# Improved Methods to Generate Spheroid Cultures from Tumor Cells, Tumor Cells & Fibroblasts or Tumor-Fragments: Microenvironment, Microvesicles and MiRNA

**DOI:** 10.1371/journal.pone.0133895

**Published:** 2015-07-24

**Authors:** Zheng Lao, Catherine J. Kelly, Xiang-Yang Yang, W. Timothy Jenkins, Erik Toorens, Tapan Ganguly, Sydney M. Evans, Cameron J. Koch

**Affiliations:** 1 University of Pennsylvania, Perelman School of Medicine, Dept Radiation Oncology, Philadelphia, Pennsylvania, United States of America; 2 Fudan University, Eye & ENT Hospital, Dept Radiation Oncology, Shanghai, China; 3 Oxford University, Gray Institute for Radiation Oncology, Oxford, United Kingdom; 4 University of Pennsylvania, Perelman School of Medicine, Penn Genomics Analysis Core, Philadelphia, Pennsylvania, United States of America; Northwestern University Feinberg School of Medicine, UNITED STATES

## Abstract

Diagnostic and prognostic indicators are key components to achieve the goal of personalized cancer therapy. Two distinct approaches to this goal include predicting response by genetic analysis and direct testing of possible therapies using cultures derived from biopsy specimens. Optimally, the latter method requires a rapid assessment, but growing xenograft tumors or developing patient-derived cell lines can involve a great deal of time and expense. Furthermore, tumor cells have much different responses when grown in 2D versus 3D tissue environments. Using a modification of existing methods, we show that it is possible to make tumor-fragment (TF) spheroids in only 2–3 days. TF spheroids appear to closely model characteristics of the original tumor and may be used to assess critical therapy-modulating features of the microenvironment such as hypoxia. A similar method allows the reproducible development of spheroids from mixed tumor cells and fibroblasts (mixed-cell spheroids). Prior literature reports have shown highly variable development and properties of mixed-cell spheroids and this has hampered the detailed study of how individual tumor-cell components interact. In this study, we illustrate this approach and describe similarities and differences using two tumor models (U87 glioma and SQ20B squamous-cell carcinoma) with supporting data from additional cell lines. We show that U87 and SQ20B spheroids predict a key microenvironmental factor in tumors (hypoxia) and that SQ20B cells and spheroids generate similar numbers of microvesicles. We also present pilot data for miRNA expression under conditions of cells, tumors, and TF spheroids.

## Introduction

Modeling the 3D environment of tumors using cells in tissue culture is known to be challenging. In particular, cell-lines derived from tumors are continuously enriched by cells having the fastest growth rates and, depending on additives and serum, for or against cells that depend on cytokines and other growth stimulatory and inhibitory factors. Use of high-density cultures, allowing 2D contact, has demonstrated modifications in radiation response that were subsequently confirmed in tumors (e.g. potentially-lethal-damage repair and modified rate of sublethal damage repair; [1, 2]). However, such cultures require frequent feeding to prevent nutrient depletion, and continue to cycle, unlike the non-cycling state commonly found for cells (usually the majority) in tumors [3]. This is an important shortcoming in their use for determining therapy response because non-cycling cells are resistant to many chemotherapy agents [4, 5]. A major step forward in tissue-culture modeling of the 3D tumor microenvironment was the discovery of multi-cell spheroids (spheroids) in suspension cultures by Sutherland and co-workers in 1970 [6]. Several of the many innovations provided by this model included 3D cell-contact effects that were shown to modify therapy response and growth properties of the cells, demonstration of drug and nutrient diffusion limitations (in common with tumors) and development of central hypoxia and necrosis [4, 5, 7].

Spheroids were originally grown in large-volume spinner cultures that required extensive maintenance and costs, but most cell lines do not form spheroids in such cultures. For this reason, alternative methods for 3D culture have been developed, most commonly by plating cells onto non-adherent dish surfaces (liquid overlay method; [8, 9]). Cell clumps were then selected and generally placed into suspension or transferred to wells of a multiwell dish. Transfer of these preformed cell clumps to suspension cultures was also not tolerated by many cell lines. Furthermore, such cultures can shed enormous numbers of cells into the medium leading to additional nutritional and feeding requirements. We showed that such daily feedings were responsible for dramatic short-term changes in spheroid microenvironment (e.g. reoxygenation; [10]). Additionally, spheroids in suspension cultures can aggregate and refragment due to interactions with each other, the spin bar or the vessel surfaces. Therefore, despite the asymmetrical growth conditions of non-stirred cultures and certain statistical requirements [11, 12], growth of individual spheroids in multiwell dishes holds many advantages, including individualized testing [13].

For more than 3 decades, our lab has utilized dissected tumor fragments (TFs) both for freezing (e.g. to initiate new tumors without intervening time in tissue culture; [14, 15]) and for short-term use to calibrate the uptake and binding of hypoxia markers such as EF5 under enforced conditions of severe hypoxia [14, 16, 17]. TFs have the advantage of containing the multiple cell-types present in tumors *in situ* and in principle should closely simulate biological and molecular properties of the original tumor tissue. When we tried to adapt them to long term cultures (e.g. by spinning or swirling) they tended to clump and then re-fragment. This is likely caused by inevitable damage at the TF surfaces (with consequent DNA-release) when they are minced from larger tissue pieces. The aggregation can be reduced but not eliminated by addition of DNA-ase (Koch, unpublished data). Several investigators have placed TFs onto non-adherent surfaces and it has been noted that the initially jagged edges become somewhat rounded over an extended period (10 days to three weeks;[12, 15]). The only consistently spherical products resulting from this process appear to be from high-grade gliomas [18].

Herein, we report the combination of these techniques by placing individual TFs into multi-well plates coated with a relatively large volume of tissue-culture-grade agarose, producing a macroscopically concave surface. For the human tumor-types investigated to date (SQ20B squamous-cell head and neck cancer and U87 glioblastoma), the randomly shaped fragments form approximate spheroidal shapes over the first 1–3 days, without outgrowth or shedding, while maintaining their multiple cell types for prolonged periods of subsequent growth. We describe the methods of their production and examples of their metabolic/molecular biology. TF spheroids may be useful in further understanding the complexity of the tumor microenvironment; indeed, under prolonged nutrient deprivation, we have observed that they can provide a 'safe-haven' for colony forming tumor cells [19, 20]. This method also provides a simple way to make mixed-cell (MC) spheroids reproducibly. This fills an unmet need often discussed in the spheroid literature [21].

## Materials and Methods

### Cell lines and culture

The human head and neck cancer cell line SQ20B, pancreatic cell line Panc1 and glioblastoma line U87, were obtained from ATCC. Human breast cancer cells (1833) and fibroblasts (MRC5) were obtained from Dr. Andy Minn, University of Pennsylvania [22]. All cell lines were cultured under standard conditions (37°, 5% CO_2_) using minimal essential media (MEM) supplemented with antibiotics, fetal calf serum (12% V/V), non-essential amino acids, 10 mM HEPES and 1 mM pyruvate [23]. Trypsinizing cells to a single-cell suspension can be complicated by their tendency to come off the dish surface in clumps. For example, epithelial cells often make extremely compact microcolonies and U87 cells form localized 'mounds'. To counteract this, we exposed monolayers to Versene (5.4 mM Na-EDTA in Hank's buffer) at room temperature for 5–15 minutes prior to trypsinizing (0.05% trypsin with 0.5 mM EDTA). Trypsin-suspended cells were treated with an equal volume of serum-containing medium to block the enzyme and were then centrifuged at room temperature (20°) 900 rpm for 9 min. After supernatant removal, the cell pellet was disrupted by forcefully tapping the centrifuge tube on a hard surface and either placed in a culture flask for future use or used for studies as described below.

### Initiation of cell- and TF-spheroids

To coat the wells of a 24-well plate, 1.5% (W/V) purified low-melt agarose (SeaPlaque—BMA) was dissolved in Hank’s saline using minimal heating in a microwave oven. After cooling to ~ 50°, 0.5 ml was added to each well of a 24-well plate. Upon cooling to RT this relatively large volume of agarose creates a substantially concave surface (agarose layer is about 1.5 mm thinner at center than edge of well). Each agarose-coated well was equilibrated via 3–4 changes of 1.5 ml medium, each for at least 4 hours in an incubator [13].

For spheroids, cells were re-centrifuged and suspended at high density in medium. About 15-20x10^3^ cells in 10 μl of medium were gently pipetted into the center of each well near the agarose surface. The cells fall along the naturally curved meniscus of the agarose to form a localized aggregate that, in many cell lines, forms a solid sphere of cells over 1–2 days. Some cell types (e.g. 1833) do not form spheroids but just maintain loose aggregates and others, such as the Panc1 follow a slower transition from disc to multilayer disk to ovoid to spheroid, taking 4–5 days. For MC spheroids, the same procedure was employed but the two cell types were both suspended at high density, then mixed (25% fibroblasts and 75% tumor cells) before adding to the well.

Animal use for this study was approved by the Institutional Animal Care and Use Committee (IACUC) of the University of Pennsylvania. For TF spheroid production, subcutaneously grown xenograft tumors grown in the flank of nude mice were removed and minced manually using crossed #11 scalpel blades into pieces approximately 2–3 mm in size. These small pieces were further minced using a dissecting microscope with the goal of producing sub-millimeter pieces. The secondary mincing was done using small drops of medium (just enough to keep tissue moist). The dissecting microscope did not fit into the sterile hood so 10x antibiotics were used for the drops. After final mincing, one piece of tissue was added per agarose-coated well, containing 1.5 ml medium as with the cells. After 1–2 days, the initially ragged tissue pieces formed nearly spherical structures and could be transferred to fresh wells using a wide-bore Eppendorf pipet tip, or simply fed as required.

### Immunohistochemistry

Cell or TF spheroids or freshly dissected tumor were frozen in OCT using small polyethylene molds. In order to confine the spheroids to a small co-planar area, diluted OCT (3 parts OCT, 1 part physiological saline) was spread in a thin film over the base of the mold and the spheroids added in a closely-spaced group. In order to prevent local dilution by the accompanying medium, spheroids were taken up with a wide mouth 200 μl pipet tip in about 50 μl of medium; the spheroid dropped to the tip in a few seconds and could be placed into the mold by simply touching the end of the tip to the OCT surface. At this point, the spheroids were thicker than the OCT film and rested by gravity on the base of the mold. The mold was then placed on a dry-ice-cooled block of aluminum, and just as it started to freeze, additional OCT was added to completely cover the spheroids [13]. Ten-micron tissue sections were obtained using a Zeiss Microm cryostat. Sections were fixed in graded alcohols at 0° (70%, 85%, 100% ethanol; 5 minutes each) and stained and imaged as described previously [24]. Normal-tissue cells were identified in the tumor, MC or TF-spheroid tissues using anti-CD31 (endothelial cells; BD Pharmingen 550274), anti-CD11b (macrophages; Abcam ab24857), anti-Ki67 (most cycling cells are tumor cells—BD Pharmingen 556003) and anti-vimentin (fibroblasts—Abcam 139855). Vimentin is not specific for fibroblasts but was present in these cells at high concentration compared with the tumor cells or other normal cells investigated to date. The anti-vimentin and anti-CD11b antibodies were self conjugated to FITC, while the others required an Alexa488-labelled secondary (Jackson 112-546-068).

### Tissue Oxygenation

The level of cellular hypoxia was estimated by incubation of spheroids with 100 μM EF5 for 4 hrs [13]. Bioreductively-produced EF5 adducts were assessed by immunohistochemistry, using Cy3-conjugated anti-EF5 antibodies, as described previously [13, 17].

### Cell Respiration

Cells were injected into stirred 10 ml vials, with temperature controlled at 37.0°. The medium used was pre-equilibrated at about 50% vacuum to lower the initial oxygen and gas concentration. The latter prevented gas bubbles from forming in the solution. The vials were sealed using inserts and oxygen sensors with standard glass tapers and respiration was measured as previously described [17, 25].

### Microvesicle (MV) collection

Microvesicles are small membrane-enclosed particles with diameters from <100 nm (exosomes) to roughly platelet size (~2000 nm) that are released by all cells. MV were prepared from the medium bathing the cells or spheroids at roughly 24 hrs following medium rinsing & replacement using fresh serum-containing medium filtered through a non-protein-binding filter (Millipore Millex GV, 220 nm). MV were isolated using methods essentially identical to those developed for plasma in patients [26]. In brief, macroscopic cellular debris was removed by centrifugation at 20° for 20 minutes at 300g (Beckman-Coulter Allegra X-22R). The supernatant was then spun at 2500g for 20 minutes (same conditions and centrifuge) to collect medium speed supernatant and pellet (MSS, MSP). The MSS was then further spun at 15,000g (30 minutes, 20°; Beckman-Coulter Microfuge 22R) giving a high-speed pellet and supernatant (HSP, HSS). All centrifugations employed swinging bucket rotors. The deceleration rate was set to zero for the Allegra and the instrument just turned off for the microfuge. This minimizes disturbance of the invisibly-small, soft pellets during centrifuge deceleration. Samples were frozen and stored at -75° as described [26].

### MV Analysis by Flow Cytometry

Sample analysis solution for the MV was isotonic NaCl and KCl, with 10 mM HEPES (pH 7.3) and 3 mM Ca, plus 1% Annexin V conjugated to Pacific Blue (BioLegend 640918) to label surface phosphotidylserine (PS). The frozen MV samples were thawed and 45μl added to 225μl of analysis solution. A BD FacsCanto instrument was set so that 3000 nm polystyrene beads were at ~100,000 on forward and side scatter (FSC,SSC). Countable particles required both a FSC and SSC greater than 250. MV were identified as a distinct group of particles, much smaller than the reference beads and with high Annexin V staining.

### miRNA Analysis

In one of the experiments using cell-derived tumors to produce TF spheroids, small RNA was isolated from SQ20B cells, freshly explanted SQ20B tumor tissue and 6-day cultured TF spheroids using the mirVana smallRNA kit (Life Technologies, Norwalk). With this kit, total RNA is fractionated into predominantly large *versus* small sizes by binding to the RNA-affinity columns at low (large RNA) followed by high (small RNA) ethanol concentrations. Small RNA concentration was first quantified using a Qubit instrument. Quality of prepared RNA samples was assessed on an Agilent 2100 Bioanalyzer using the Small RNA Kit. Quantification of miRNA was estimated by measuring the concentration of nucleic acids in the 10–40 nucleotide range using the Agilent 2100 Expert Software. Based on this value, ~25ng of miRNA per sample was used as input for sequencing library preparation using Ion Total RNA-Seq Kit v2 (Life Technologies). Each sample library was tagged with a unique DNA barcode to facilitate pooling of multiple samples into one sequencing experiment. Pooled samples were sequenced on an Ion Torrent Personal Genome Machine using 316 and 318 chips and 200 base sequencing chemistry. Basecalling and quality filtering of sequence data was performed automatically by the Ion Torrent Suite software. Sequence data was exported in FASTQ format for further analysis as recommended by an Ion Torrent RNA-Seq white paper [27]. Sequence reads were trimmed using the FASTX-Toolkit [28]. Trailing 3' bases with PHRED scaled quality values below 17 were removed, and any reads shorter than 17bp were discarded to ensure high mapping specificity. Any reads exceeding 35bp were trimmed from the 3' end to that length. Trimmed reads were first aligned to a reference containing rRNA and tRNA sequences retrieved from NCBI RefSeq database [29] and UCSC table browser [30] respectively, using the Bowtie v. 1.1.1 aligner [31]. Reads that did not align to tRNA or rRNA reference sequences were then aligned to the human subset of mature miRNA sequences in miRBase version 21 [32] using the SHRiMP2 aligner [33] in miRNA mode with default parameters. After alignment the number of reads aligning to each miRNA detected were counted using a custom bash script and output in CSV format.

## Results

**Fig 1:**
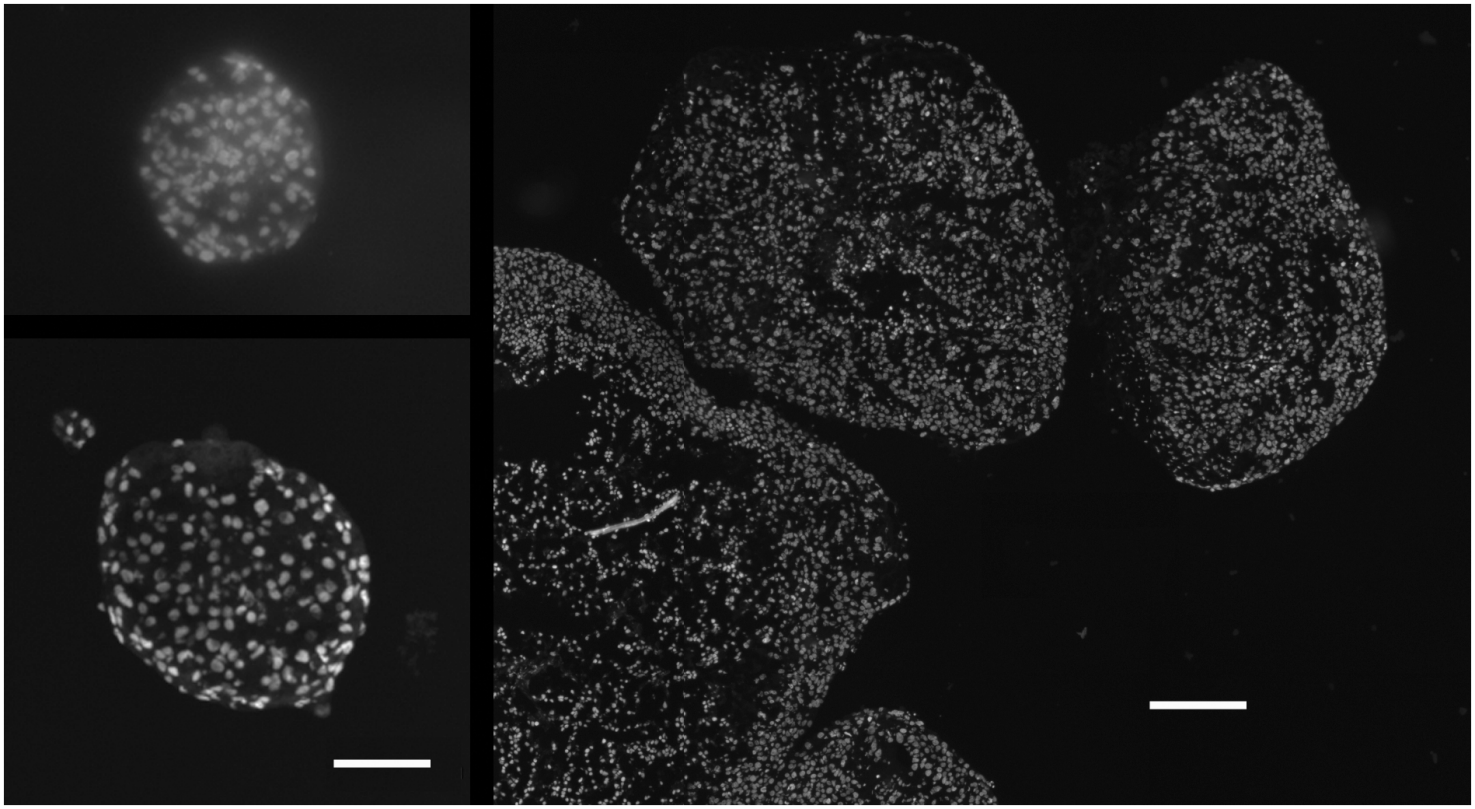
Cross sections of various types of SQ20B spheroid. Spheroids from tumor-cells only were very small (upper left), those from mixed-cells (with MRC5) somewhat larger (lower left) and from tumor-fragments much larger still (right; all nuclei stained with Hoechst 33342). The larger TF spheroids developed central necrosis (absent or pyknotic nuclei) with a relatively thin rim of viable cells. The scale bars represent 100 μm for the two left panels and 250 μm for the right panel.

**Fig 2:**
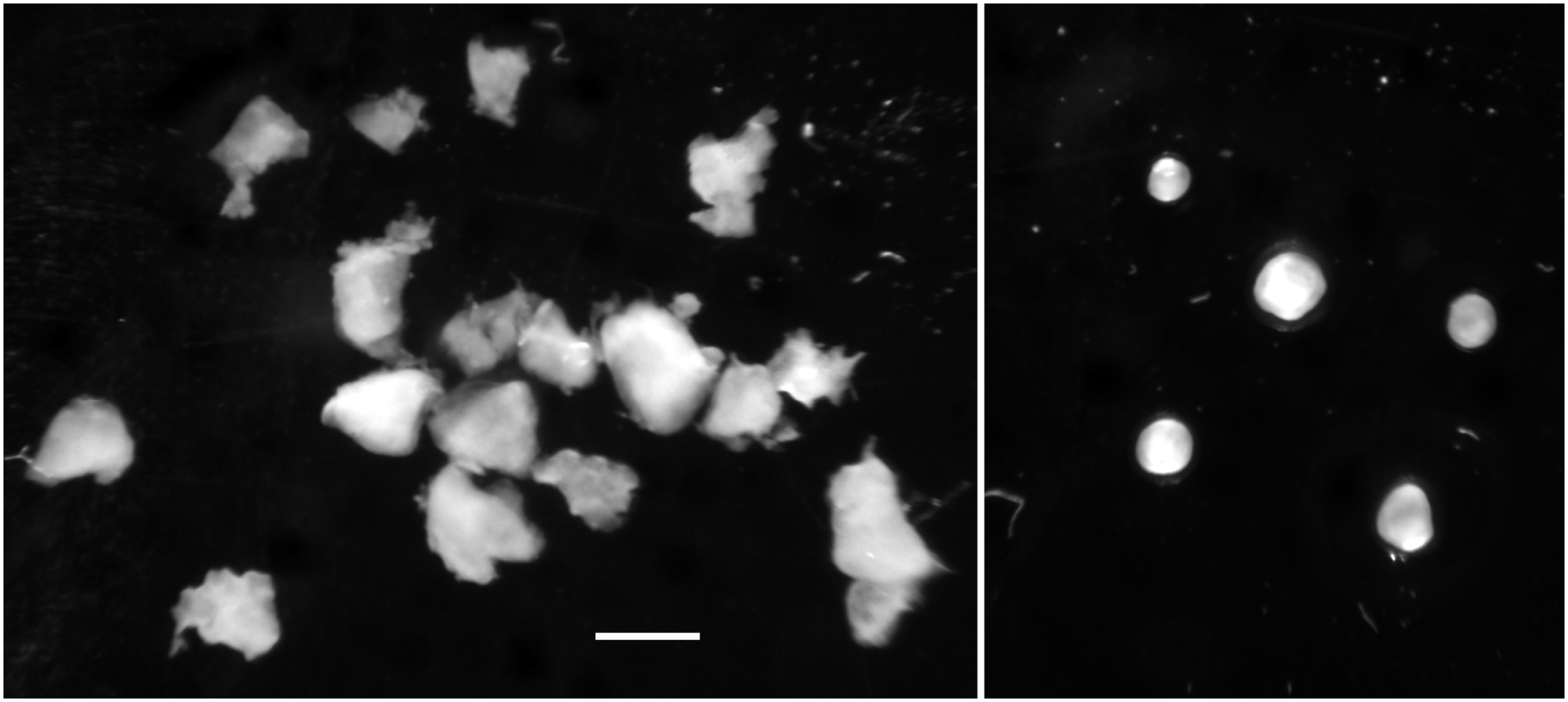
Tumor fragments to spheroids. Freshly-cut tumor fragments from SQ20B tumor (left panel) formed spheroids within 2–3 days (right panel). (representative of several experiments—scale bar is 1000 μm).

SQ20B spheroids, irrespective of their initiation method, formed only very small spheroids *in vitro*, with diameters limited to 200 μm or so (see also [34]). Spheroids did not form at all in spinner or swirled culture. Larger-sized spheroids were formed when human fibroblasts (MRC5) were mixed with the SQ20B cells, even though the MRC5 cells themselves also formed only very small spheroids (Fig 1). It is of interest that the 1833 breast cancer cells did not form even small spheroids in our liquid overlay (only loose aggregates), but did so when mixed with MRC5 fibroblasts (Table 1). Fibroblasts are not generally required [21] since U87 glioblastoma and Panc1 adenocarcinoma cells made very large spheroids even without the addition of stromal cells (Table 1) which is consistent with the general use of the liquid overlay technique. In contrast to the above complexities, SQ20B tumor fragments placed into the liquid overlay culture quickly (~2 days) formed large TF spheroids that remained viable for two to three weeks in culture (Figs 1 and 2).

**Table 1:** Characteristics of cells and spheroids used in this study.

Cell Line	Origin	Spheroid	Rate	EF5 Binding	TF Spheroids
SQ20B	H&N Carcinoma	Yes^1^ ^,^ ^3^	Fast	Yes^4^	Yes^5^
U87	GBM	Yes	Fast	No	Yes
Panc1	Pancreatic Adeno	Yes^2^	Slow	Yes	ND
1833	Breast Carcinoma	No	NA	No	ND
MRC5	Normal Fibroblast	Yes^3^	Fast	No	NA

Notes:

^1^. Larger with addition of MRC5 fibroblast cells;

^2^. Spheroid formation goes through observable changes from disc to sphere that take about 5 days;

^3^. Spheroids are small and have limited growth potential;

^4^. EF5 binding was only seen in MC and TF spheroids—hypoxia in spheroids was only observed centrally.

^5^. Although SQ20B and U87 TFs both underwent rapid transformation to spherical shape, central hypoxia and necrosis in the former limited any growth, so their size reached a maximum at about 6 days and then declined with time. U87 TF spheroids continued to grow and could reach 1.5 to 2 mm in diameter. NA means non-applicable, ND means not done.

**Fig 3:**
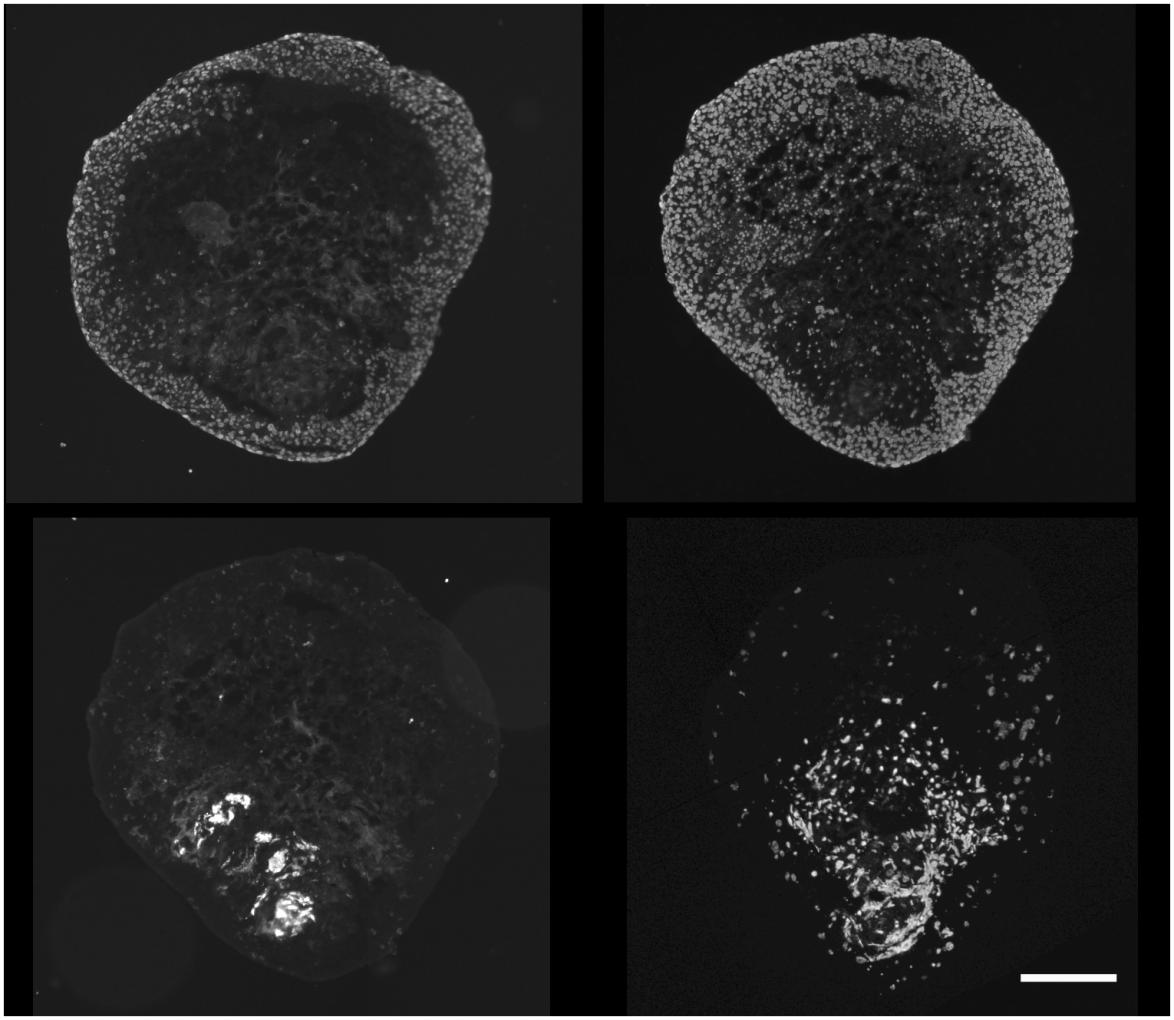
Stromal cells in TF spheroids. 6-day TF spheroids retained host tissue components, some of which migrate to characteristic positions in the spheroid. Macrophages (CD11B positive) migrated to the periphery (upper left), and, as in this example sometimes comprised a sizeable fraction of the total cells (indicated by Hoechst 33342 staining of nuclei) (upper right). CD31 positive endothelial cells remain grouped together (lower left) while vimentin positive cells tended to congregate within and around the central hypoxic/necrotic zone as well as the periphery (lower right. (representative of several TF spheroids from 2 preparations—scale bar is 250 μm).

The stromal characteristics of the SQ20B TF spheroids retained several characteristics of the tumors from which they are derived (Fig 3). For example, endothelial cells remained self-associated since they derive from intact vessels and we have no evidence for their subsequent migration. Cells staining for vimentin in the TF spheroids formed internal colonies, tending to be associated with central necrosis but also migrated to the periphery. In the TF spheroids, macrophages migrated to the outer portion of each sphere (Fig 3).

**Fig 4:**
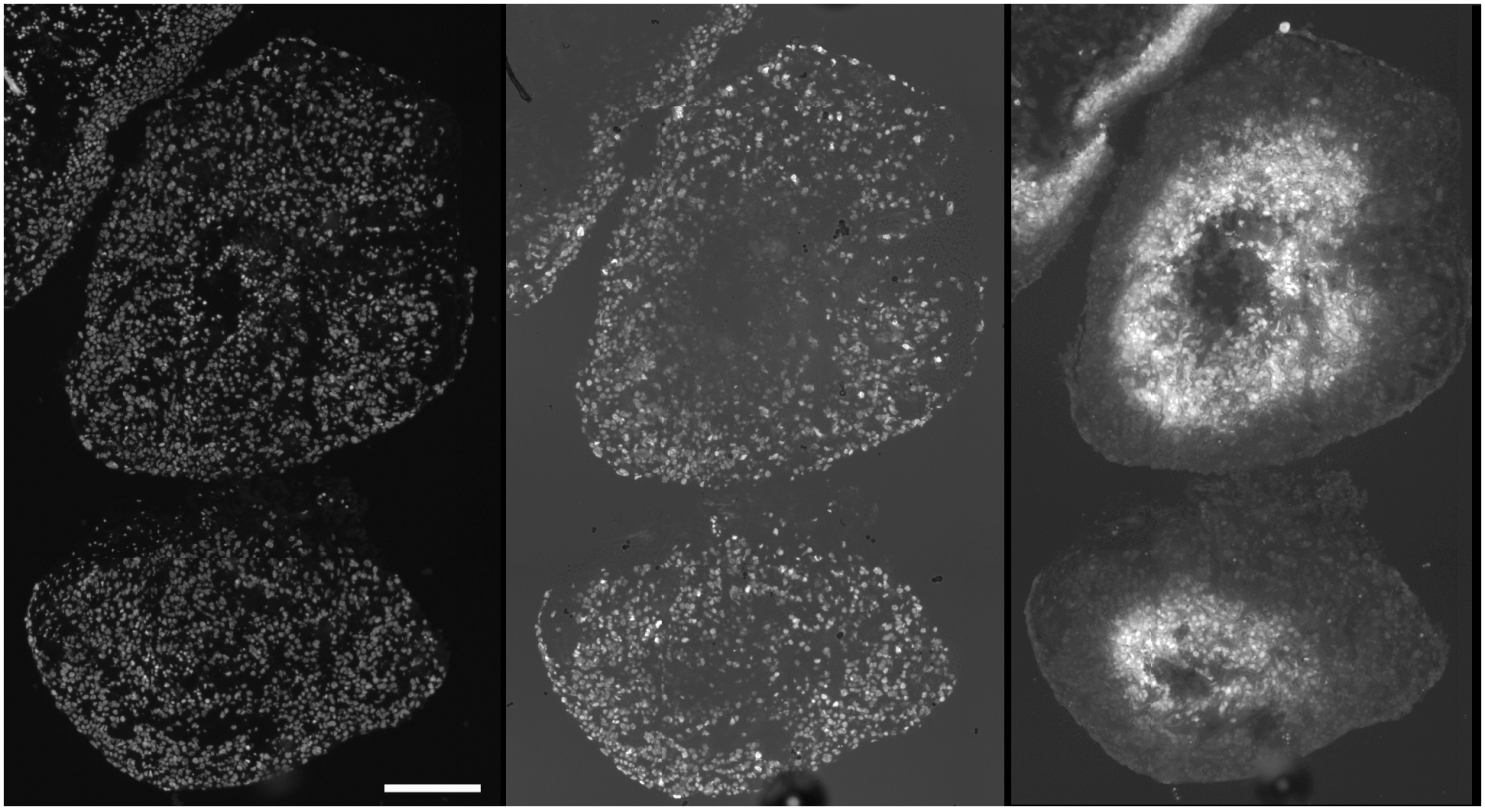
Cell cycling and EF5 binding in SQ20B TF spheroids. Cycling Ki67 positive cells were peripherally localized (central panel), in contrast to the inverse location of EF5 binding cells (right panel). Hoechst 33342 staining of all cell nuclei shown in the left panel. For the larger, more necrotic TF spheroids (small portion seen in upper left of each panel), the EF5 positive cells lined the inside of the viable rim, since EF5 is only metabolized by viable cells (scale bar 250 μm). Ki67 positive cells were also found peripherally in U87 spheroids of all types, but no EF5 binding was observed (data not shown).

Ki-67 positive cells were confined to the external portion of viable rim for SQ20B TF spheroids. Conversely, EF5 binding occurred in the central area of these spheroids (Fig 4). SQ20B cell spheroids were too small to form central hypoxia, but small regions of EF5 binding were found in the MC spheroids (data not shown).

**Fig 5:**
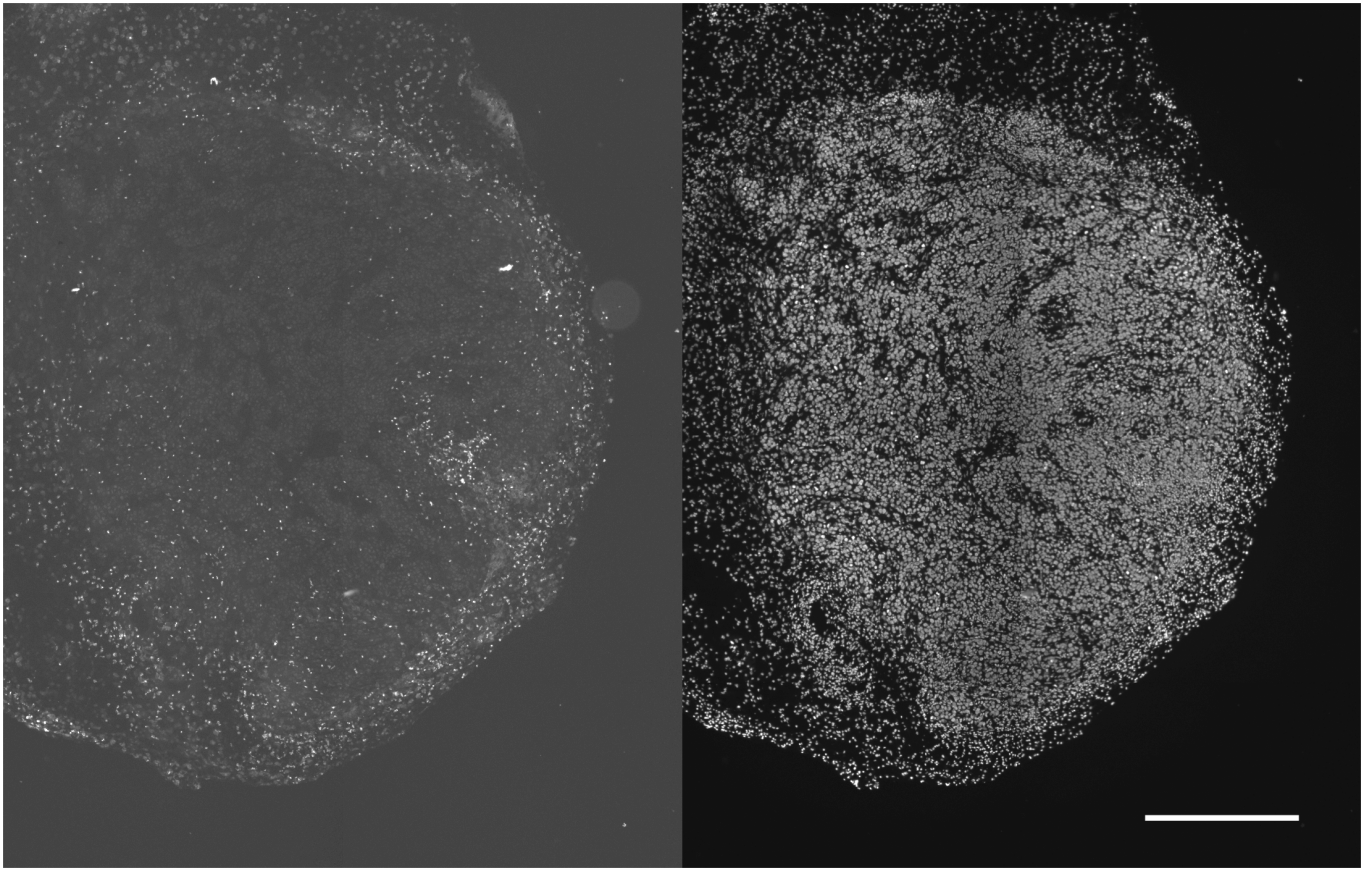
CD11b cells in SQ20B subcutaneous tumors. CD11b positive cells were minimally found in the tumor interior (left panel). Total cells illustrated in the right panel, showing Hoechst 33342 nuclear staining (scale bar 0.5 mm). The lower density of nuclei along the left edge of the section showed the transition from tumor to normal tissue.

**Fig 6:**
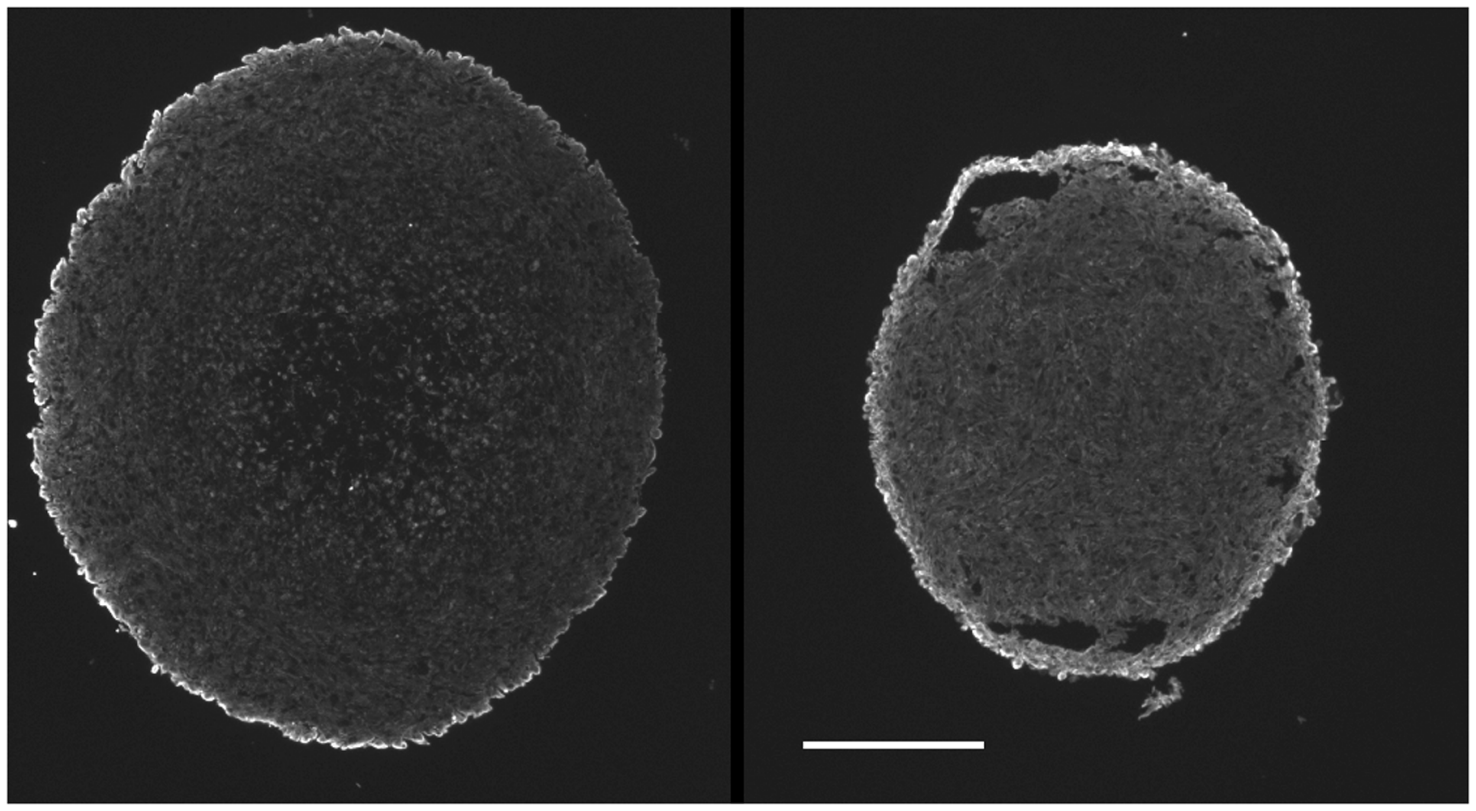
MRC5 fibroblasts form a surface capsule in MC spheroids. In 10-day mixed cell spheroids, vimentin positive fibroblasts migrated to form a surface capsule (left U87+MRC5; right Panc1+MRC5—scale bar 0.25 mm). No surface vimentin staining was observed for spheroids consisting solely of either tumor cell type (data not shown).

**Fig 7:**
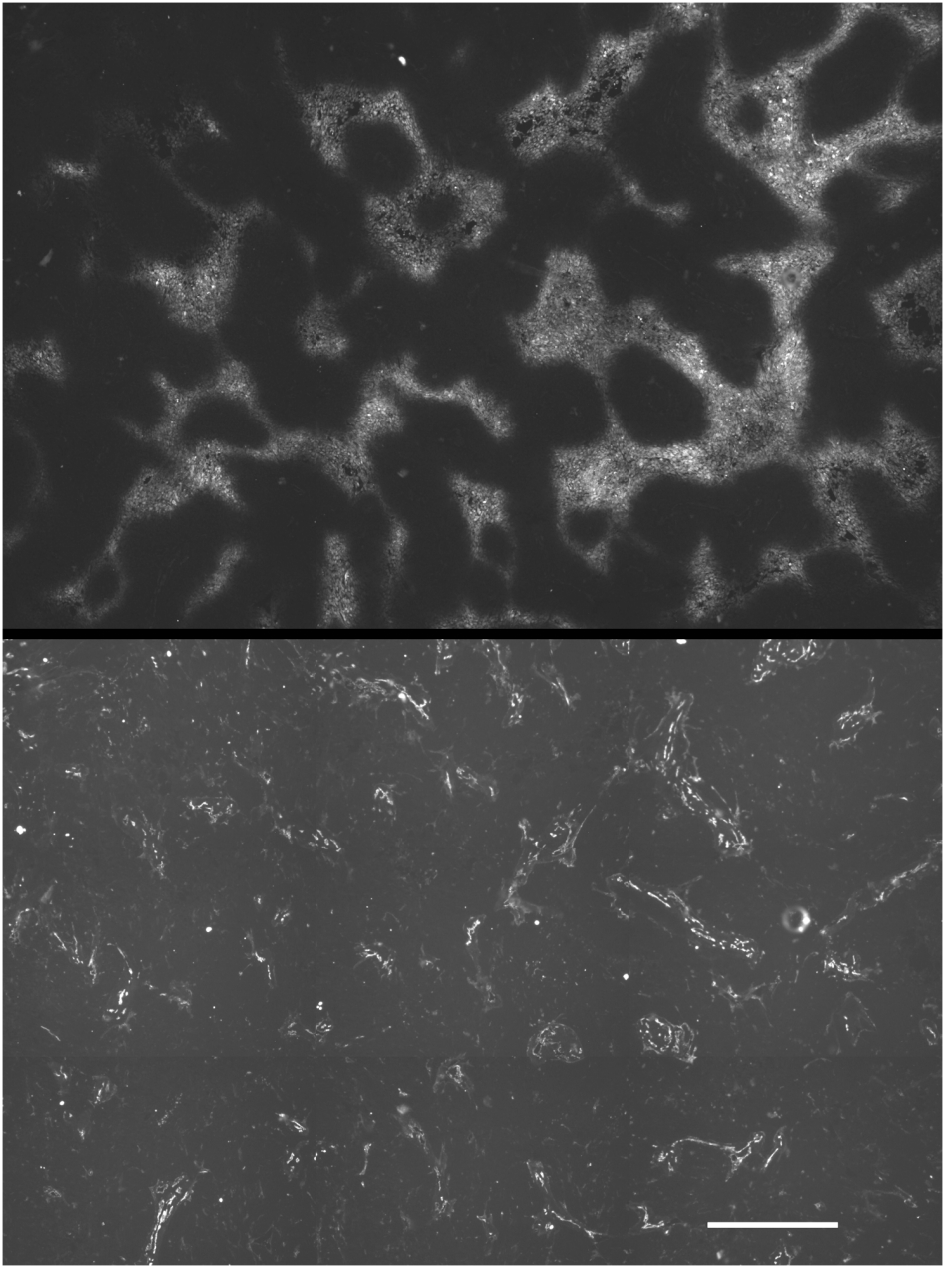
SQ20B tumors were hypoxic. SQ20B tumors contained EF5 binding (upper panel) which was inversely correlated with the position of blood vessels (lower panel). (scale bar 500 μm).

For the SQ20B tumor from which the TF spheroids were derived there were many more CD11B positive cells in the tumor periphery than in its interior (Fig 5). Similarly vimentin positive cells were found in interior colonies and the exterior stroma (data not shown). The position of vimentin positive cells was most clearly defined for the MC spheroids. Regardless of tumor-cell type, vimentin positive staining was always observed as a peripheral capsule (Fig 6)—this type of binding pattern was never observed in spheroids derived solely from tumor cells (data not shown). SQ20B subcutaneous tumors have varying degrees of hypoxia as evidenced by EF5 binding. Detailed investigation of more than 10 tumors showed that more than half contained moderate to severe hypoxia [17]. Tumor vasculature had a strongly negative correlation with EF5 binding and vessels were typically found at the center of the non-EF5 binding regions (Fig 7)—this is consistent with diffusion-limited hypoxia [24].

**Fig 8:**
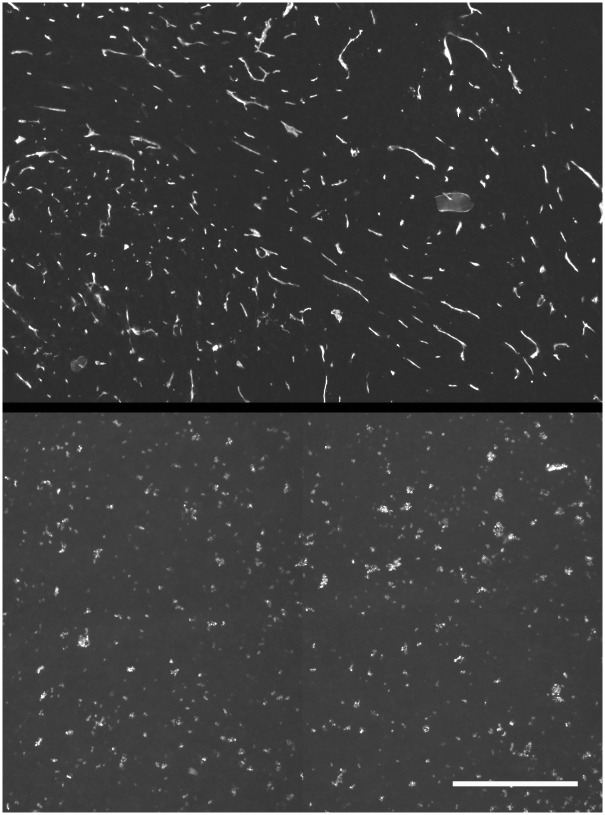
U87 tumors were aerobic. U87 tumors had a much higher density of vessels (upper panel) than did SQ20B tumors which explains their lack of EF5 binding. The lack of hypoxia was associated with a relatively uniform distributions of macrophages (CD11b positive; lower panel) but it is not known if this was causally associated (scale bar 500 μm).

**Fig 9:**
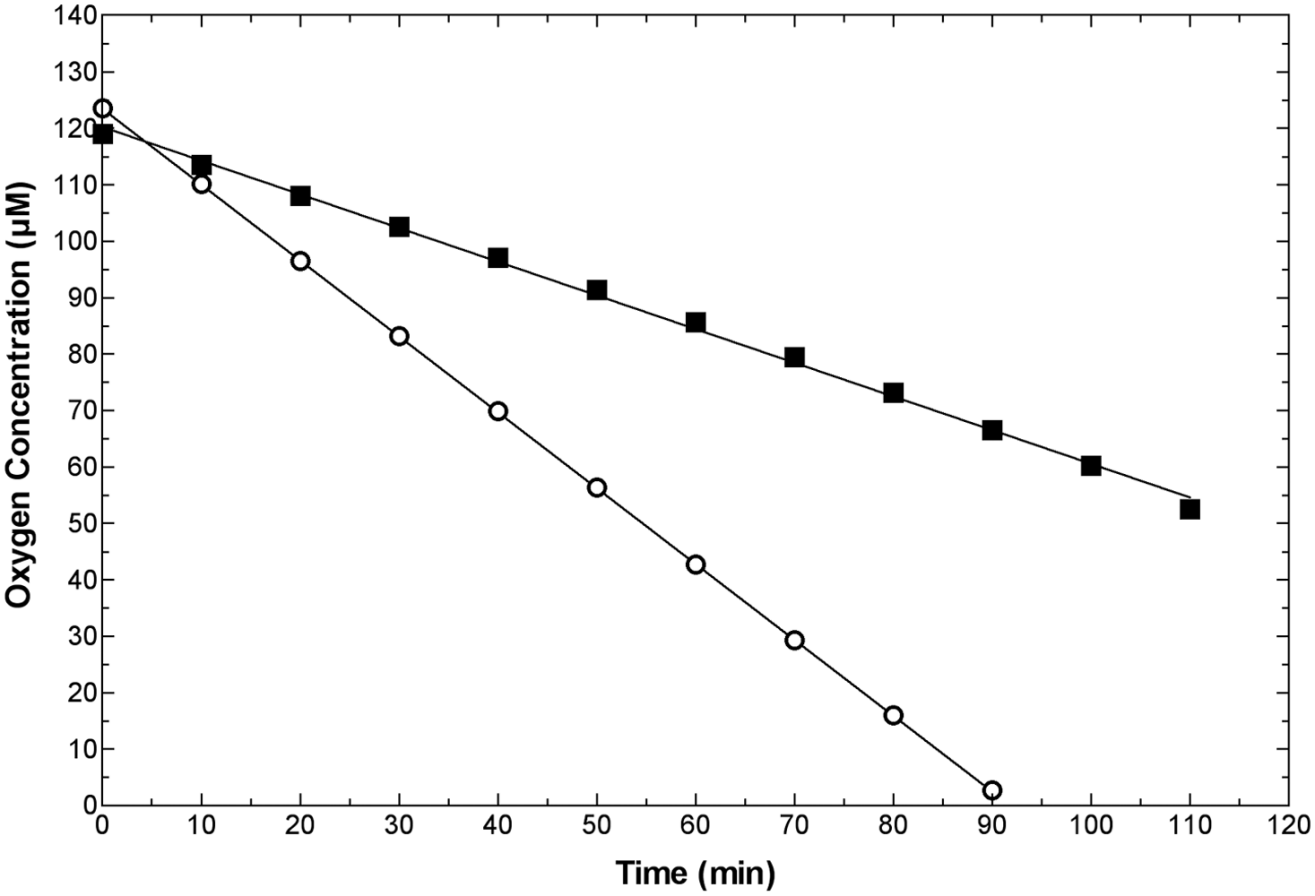
Respiration rates of U87 *versus* SQ20B cells. The respiration rate of U87 cells (closed squares) was about one third that of SQ20B cells (open circles). pO_2_ was monitored as a function of time in a closed, stirred 10.0 ml vial containing medium at 37°. Cell numbers were similar for this experiment, but cells were added based on their pellet weight to avoid counting issues related to multiplicity/cell-clumping. Thus, each vial contained 10 mg of cell mass. These data are representative of 3 experiments, giving respiration rates for U87 and SQ20B cells (respectively) of 2.7±0.5 and 8.6±3.2 (x10^-17M/cell/sec).

The U87 glioma line has somewhat different properties, as indicated in Table 1, and was capable of forming large spheroids (>1mm diameter) without the addition of stromal cells. Nevertheless, TFs from this tumor formed TF spheroids in less than 2 days and then underwent substantial additional growth. Typically, both types of large spheroid required feeding at one or two day intervals to prevent nutrient depletion. EF5 binding was not observed in U87 xenograft tumors. This might be expected from this cell line’s high endogenous production of vascular endothelial growth factor (VEGF—see [35]) and indeed the tumors were very highly vascular (Fig 8). We have looked at more than 12 subcutaneous U87 tumors with no observed EF5 binding. U87 cell and TF spheroids demonstrated little to no EF5 binding when cultured *in vitro*, which was surprising considering their large size. This unexpected finding appears to result from a highly decreased respiration rate in this cell (Fig 9). Perhaps due to its high vascularity and lack of hypoxia, macrophages were uniformly distributed throughout this tumor (Fig 8) in contrast to what was found for the SQ20B tumors.

**Fig 10:**
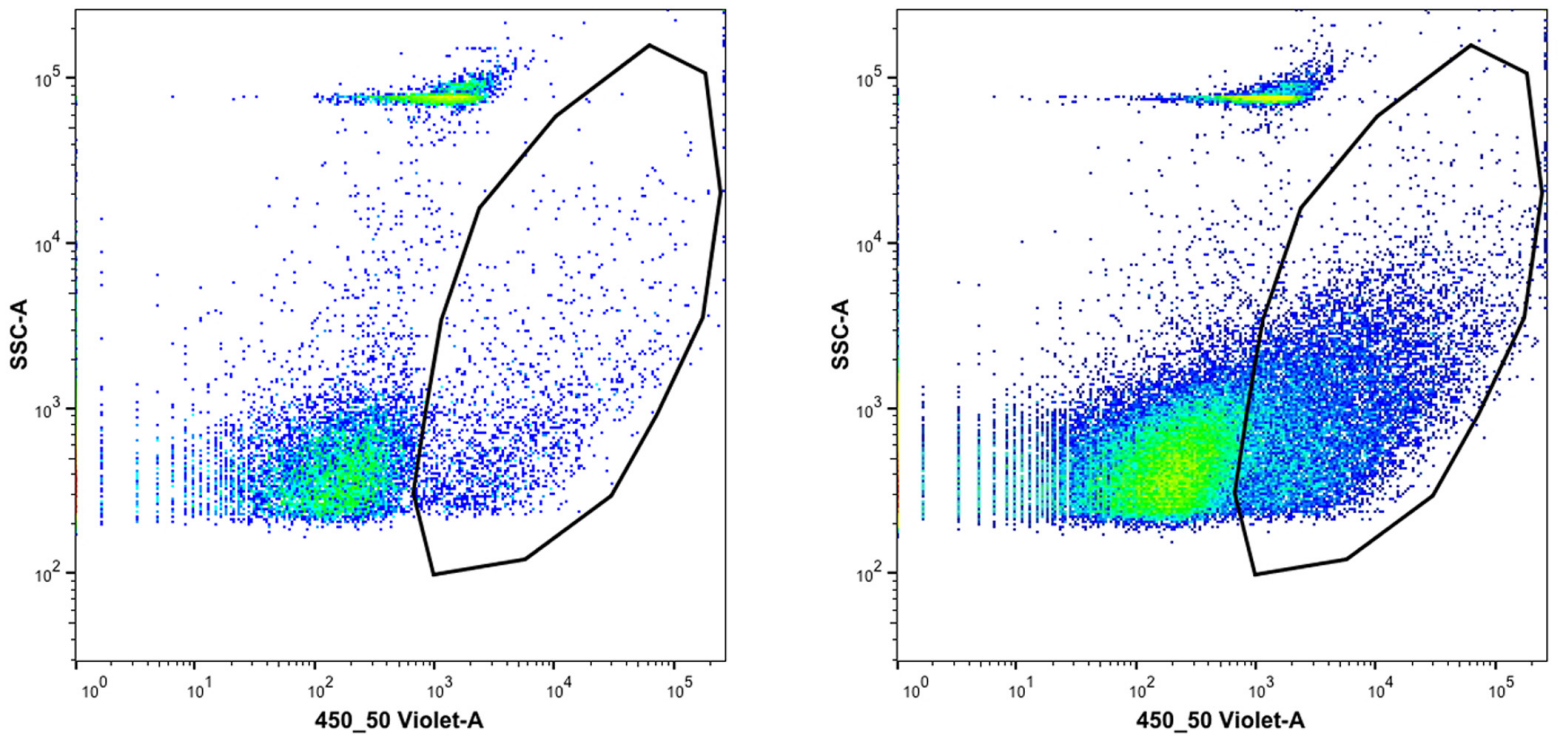
Microvesicles from tissue culture medium 24 hr following medium change to SQ20B cells. Typical output from the flow cytometer showed two particle populations. Those with the polygon surrounding them had high phosphotidyl-serine content, as measured by Pacific Blue labeled Annexin V, while the rest did not stain for this surface marker: left panel, low speed supernatant, right panel medium speed pellet. There were eight-fold more MV in the pellet, which was expected based on the final volumes assuming collection of almost all MV. The fraction of Annexin V positive particles was about 0.09 for the left panel and 0.18 for the right panel.

**Fig 11:**
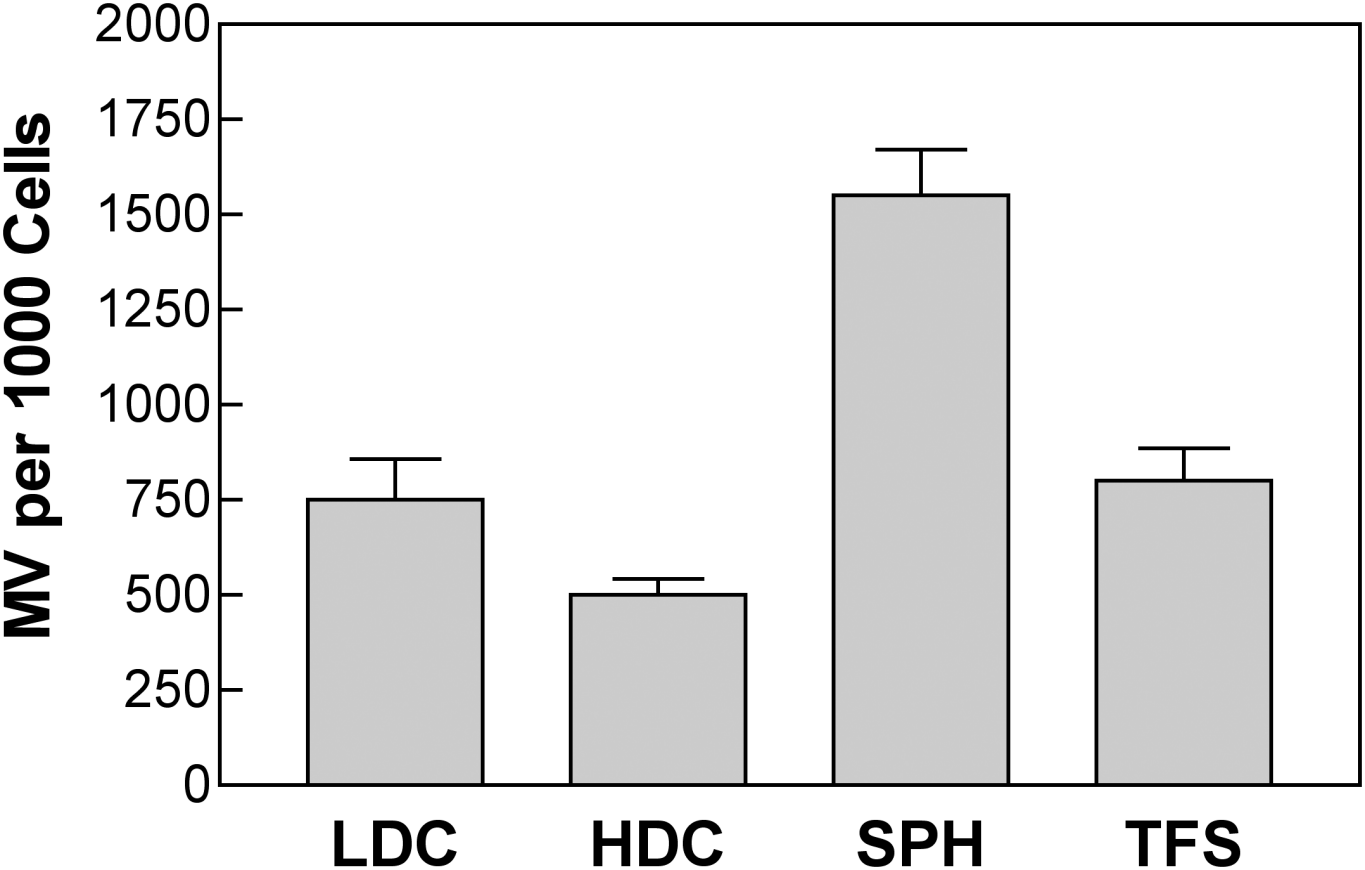
MV per 1000 cells. Quantification of MV production per 1000 SQ20B cells over a 24 hr period under four conditions of growth: LDC—low-density cells; HDC—high-density cells; SPH; tumor-cell spheroids; TFS—TF spheroids (error bars SD of 3 replicates).

Microvesicle release from 3-D tissue cultures has not previously been demonstrated, but this should be possible since tumor-derived microvesicles are found within the circulating blood [26]. In fact, considering all the cells in the spheroid, not just those at the surface, cell and TF spheroids produced the same or more MV than did similar numbers of cells in culture. At present we do not know whether this unexpectedly high release from the 3-D cultures represents a higher rate of production by the surface cells, an ability of the MV to diffuse out of the 3-D structure, or a new effect of 3D growth (Figs 10 and 11).

In a pilot test for the similarity of miRNA expressed in 2-D cell culture, tumors and TF spheroids, miRNA was sequenced from SQ20B cells (10^6^), cell-derived tumor (3 mg piece of fresh tissue) and tumor-derived TF spheroids (4 of the 6 day TF spheroids). Since the sequences of corresponding murine and human miRNA are often identical it is not usually possible to isolate the stromal from tumor components of the tumor and TF spheroid tissue based on their miRNA sequences. The 50 most abundant miRNA’s for cells were rank-ordered and the corresponding ranks listed for tumors and TF spheroids (Table 2). All miRNA’s found (>1000) as well as their ranks and number of sequences, normalized per million reads, is provided in S1 Table.

**Table 2:** Ordinal ranking of most abundant SQ20B cellular miRNA’s and corresponding ranks for SQ20B tumor and its derived TFS (6-day).

GENE	Cells	Tumor	TFS	GENE	Cells	Tumor	TFS
hsa-miR-205-5p	1	2	3	hsa-miR-18b-5p	26	54	36
hsa-miR-21-5p	2	1	1	hsa-miR-18a-5p	27	55	36
hsa-let-7a-5p	3	3	2	hsa-miR-130a-3p	28	53	32
hsa-let-7c-5p	4	10	4	hsa-miR-29c-3p	29	17	35
hsa-miR-30a-5p	5	16	25	hsa-miR-20a-5p	30	48	33
hsa-miR-19b-3p	6	8	24	hsa-let-7b-5p	31	36	26
hsa-miR-19a-3p	7	9	28	hsa-miR-24-3p	32	32	20
hsa-miR-23b-3p	8	6	6	hsa-miR-106a-5p	33	56	27
hsa-miR-23a-3p	9	7	5	hsa-let-7d-5p	34	43	30
hsa-miR-30d-5p	10	18	29	hsa-miR-125a-5p	35	38	17
hsa-miR-103a-3p	11	12	18	hsa-let-7e-5p	36	49	38
hsa-miR-107	12	12	18	hsa-miR-31-5p	37	33	21
hsa-miR-17-5p	13	24	12	hsa-miR-93-5p	38	62	31
hsa-let-7f-5p	14	11	11	hsa-miR-29b-3p	39	31	43
hsa-miR-27b-3p	15	4	9	hsa-let-7g-5p	40	25	47
hsa-miR-27a-3p	16	5	9	hsa-miR-26a-5p	41	34	13
hsa-miR-30c-5p	17	20	22	hsa-let-7i-5p	42	26	48
hsa-miR-30b-5p	18	19	23	hsa-miR-26b-5p	43	35	15
hsa-miR-221-3p	19	42	44	hsa-miR-106b-5p	44	46	53
hsa-miR-222-3p	20	22	41	hsa-miR-200a-3p	45	30	40
hsa-miR-16-5p	21	14	14	hsa-miR-141-3p	46	29	39
hsa-miR-195-5p	22	15	16	hsa-miR-7-5p	47	76	79
hsa-miR-200b-3p	23	27	8	hsa-miR-320a	48	68	46
hsa-miR-200c-3p	24	28	7	hsa-miR-15b-5p	49	40	58
hsa-miR-3607-5p	25	23	52	hsa-miR-181b-5p	50	41	54

## Discussion

In this report we demonstrate a simple method that allows the production of tumor cell, mixed-cell and tumor-fragment spheroids. We use this model to demonstrate, for the first time, MV release from 3D cultures. We also find generally similar levels of the most abundant miRNA for cells and derived tumor and TFS.

Use of quantities of agarose large enough (0.5 ml) to make a macroscopically concave surface in 24-well plates appears to be the key step in facilitating the formation of MC and TF spheroid types. We had previously shown that one can localize cells to the center of a flat dish by simply inoculating them there using a relatively small volume of cells at high cell density [36]. Adapting this approach to the rapid initiation of cell and MC spheroids by using smaller numbers of cells at similar density allows a highly localized and uniform mixture of both cell types, and the concave surface shape appears to enhance the overall process. This allows either larger spheroids (SQ20B) or their formation by cells that would not normally form spheroids (1833 breast cancer cells) and very consistent proportions of the two cell types in MC spheroids. MC spheroids have previously been characterized by highly inconsistent proportions of host *versus* tumor cells [37, 38]. The same method allows the formation of single-cell-type spheroids with excellent reproducibility and rapidity. This was particularly true for cells that aggregate slowly, such as the Panc1 cells indicated herein. This contrasts with a recent report showing that Panc1 cells do not form spheroids with standard liquid overlay methods [39].

The role of the concave agarose in allowing the rapid formation of TF spheroids is less clear. However, we confirm the much longer times required when trying to initiate these in groups on a flat agarose surface [12, 38]. It is possible that use of an individual well per TF spheroid is critical to prevent interactions with neighboring fragments, as would occur in grouped cultures. Certainly, this method has completely solved our former problems with trying to grow these in swirled or stirred suspensions immediately following their dissection from a solid tumor mass. The ability to make consistent MC spheroids, combined with clear survival of host cells in the TF spheroids suggests that it may now be possible to dissect specific interactions between host and tumor cells in various combinations.

Investigators in Norway have found that TF spheroids from patients with glioblastoma retain the genetic characteristics of the original tumors and have also found that it is possible to store them without observed changes in their characteristics [15, 18]. This considerably extends our former demonstration of the ability to freeze TF, though using a different freezing technique, in order to prevent the expense of continuous *in vivo* passage. It also allows the possibility of doing extensive animal experiments over time while starting all tumors from a common source [14]. The GBM tissue fragments appear uniquely suited to rapid TF spheroid formation even in grouped cultures [18].

Most of the antibodies used for the immunohistochemical studies in this report are highly specific with the possible exception of anti-vimentin. Vimentin is known to be produced by multiple cell types and, while strongly positive for fibroblasts, cannot be considered absolutely specific for this cell type. For the tumor cell lines used however, vimentin staining is very low and is only obvious for the MC spheroids. Similarly, we have observed no overlap of staining for the antigens investigated (CD31, CD11b, vimentin).

With the known modifications in drug response due to 3D contact (typically increased drug therapy resistance), an important potential use for spheroid systems is in drug screening. For this purpose, use of spheroids in multiwell dishes is an important advantage, despite the asymmetrical growth conditions [13]. Since statistical requirements suggest the need for multiple spheroids per assay [11] this may require a similar number of independent wells to prevent spheroid-to-spheroid interactions [12]. To scale up the overall process, microfabrication and microfluidics approaches have been suggested but these may ultimately limit the size of spheroid or its tumor-simulating environmental state. For example, Yeon *et al* have suggested the use of predefined hemispherical microwells to mediate the rapid formation of spheroid shape [39]. However, this system has a severe asymmetry in that the lower half of the spheroid has no access to nutrients and drugs and does not accommodate spheroid growth beyond the size of the well. Hence, its diffusion characteristics may have some characteristics in common with planar multilayer systems [40, 41].

This is the first report of MV release from 3D cultures and it is interesting that the numbers of MV are as large or greater than those produced by the same number of cells in monolayer (Fig 11). Our initial expectation was that only the surface cells would be releasing MV of the size investigated here (>300 nm diameter). Thus, it is possible that cells on the spheroid surface are releasing far more MV than would be expected by individual cells, that the MV can be released and escape from the entire volume of the spheroid by a mechanism that is not presently understood or that the 3D microenvironment itself stimulates the production of MV. Very recent studies have suggested an important role of mixed-cell cultures in promoting therapeutic resistance changes induced by exosomes [22]. Since the 3D spheroid environment is also associated with therapy resistance it will be interesting to find out if the mechanisms have common factors. Solving the technical problem of producing consistent MC spheroids (tumor cells and fibroblasts) and TF spheroids is an essential first step to answering this important question.

None of the cell types studied herein produced MV that have strong surface markers other than PS, although we have only looked for tissue factor, tetraspanin, E-cadherin and EpCAM (data not shown). Thus, we cannot yet determine whether the MV produced are coming proportionately from all included cell types in the MC and TF spheroids. From our *in vivo* studies, most PS positive MV are also strongly positive for CD41 (platelets) but we would not expect significant remaining blood components for the TF spheroids [26].

Along the same line, we surmised that inhibition of cell cycling (compared to 2D cultures) in spheroids of all types might involve enhanced production of specific miRNA species [39]. Although much more work would have to be done to determine the possible effects of miRNA's expressed at minority levels (see S1 Table), our pilot data shows great similarity between the 3 conditions. The 50 most abundant miRNA's expressed in SQ20B cells are found in the 76 most abundant miRNA’s in an SQ20B tumor, and the 79 most abundant miRNA’s from its derived TFS (Table 1). Unfortunately, many of the murine and human miRNA sequences are identical and thus do not provide informative differentiation between stromal and tumor cells present in xenografts and TF spheroids (work in progress).

In terms of critical microenvironmental factors such as hypoxia production, the TF spheroids provided a valuable resource for testing differences between the U87 and SQ20B tumor types. Despite the ability of U87 cells to form very large spheroids, neither they nor their TF counterparts formed central hypoxia, although reduced cell densities were often observed in that location. Interestingly, similar findings were observed in the corresponding tumors—U87 tumors consistently demonstrated no EF5 binding whereas SQ20B tumors generally demonstrated substantial hypoxia (Figs 7 and 8). This is not due to the inability of EF5 to be bioreductively metabolized by U87 cells since cube reference binding and binding kinetics *in vitro* are high for this cell [42]. The simplest explanation for our observation is the dramatically reduced rate of respiration found for the U87 compared with SQ20B cells (Fig 9). Compared with most other cells investigated, the respiration rate for the SQ20B cells (~9x10^-17^ moles/cell/sec) is on the high side, whereas that of the U87 cells (~3x10^-17^ moles/cell/sec) is very low, especially considering their large size [17, 43]. We will be testing whether the respiratory controls recently elaborated by one of the co-authors also apply in this case [13]. Having described a facile and rapid method for their production, it would certainly be interesting if the properties of TF spheroids *in vitro* could signal whether or not a particular tumor was likely to form hypoxia *in vivo*.

The original promise for using spheroids and particularly TF spheroids to assess a patient's individualized therapy response seems now possible in some types of brain cancer [44] and head and neck cancer [12]. In this report, we show physiological similarities between the distribution of host cells in tumors and TF spheroids. In particular, the exclusion of macrophages from the central regions of SQ20B tumors is recapitulated by their migration to peripheral regions of TF spheroids (Figs 4 and 5). This agrees with an overall downregulation of immune elements in hypoxic regions of 9L gliosarcoma tumors in rats [45]. Our observed lack of such migration in U87 TF spheroids is consistent with the lack of hypoxia seen in this model (Fig 8).

The ability to form mixed-cell spheroids with consistent properties may find similar utility in dissecting the role of individual host cell types in the tumor environment. This has been considered an important but unmet need in the literature [37]. Although we have only shown this for tumor cells and fibroblasts, the method will hopefully be generally extendible to tumor cells paired with several different stromal cell types and possibly with more than 2 types of cells simultaneously.

## Conclusions

Methods are detailed to rapidly and reproducibly produce spheroids derived from tumor cells, tumor cells plus fibroblasts, and tumor-tissue fragments. These allow detailed biological and molecular studies to be performed *in vitro* under conditions closely simulating the *in vivo* state.

## Supporting Information

S1 Table Absolute reads and normalized reads per million for all miRNA’s sequenced for cells, tumor fragment and TFS; order based on cell data. (XLS)
